# Synergistic effects of combined motor and language interventions on stroke rehabilitation: a holistic approach

**DOI:** 10.3389/fnhum.2024.1454491

**Published:** 2024-11-21

**Authors:** Reihaneh Saber-Moghadam, Afsaneh Zeinalzadeh, Jamshid Jamali, Mohammad Taghi Farzadfard, Davood Sobhani-Rad

**Affiliations:** ^1^Department of Speech Therapy, School of Paramedical and Rehabilitation Sciences, Mashhad University of Medical Sciences, Mashhad, Iran; ^2^Department of Physiotherapy, School of Paramedical and Rehabilitation Sciences, Mashhad University of Medical Sciences, Mashhad, Iran; ^3^Department of Biostatistics, School of Health, Social Determinants of Health Research Center, Mashhad University of Medical Sciences, Mashhad, Iran; ^4^Department of Neurology, Faculty of Medicine, Mashhad University of Medical Sciences, Quaem Medical Center, Mashhad, Iran

**Keywords:** stroke, aphasia, Broca, rehabilitation, language, motor, hand movement

## Abstract

**Background:**

Stroke patients typically suffer from a range of symptoms, such as motor and language impairments, due to shared neural networks. The recovery process after stroke is intricate and requires a comprehensive approach. While previous studies have investigated the motor and language interventions independently, this study aimed to explore the relationship between these domains and compared the effectiveness of individual interventions versus their combined use.

**Methods:**

We divided 45 stroke patients into three groups: Speech and Language Therapy (SLT) group; Arm Ability Training (AAT) group; and consecutive combination of SLT and AAT group. Participants attended 40-min sessions three days a week for three weeks. Standardized assessments, including picture naming test, syntactic comprehension test, and Test d’Evaluation des Membres Supérieurs de Personnes Âgées (TEMPA) test, were conducted pre-and post-treatment and during the first and second weeks of the intervention.

**Results:**

Within-group comparisons demonstrated a significant enhancement in test scores for all groups post-intervention compared to pre-intervention (*p* < 0.05). Between-group comparisons revealed significant differences (*p* < 0.05) in performance on the picture naming test during the first week, the syntactic comprehension test in the second week, the functional rating subscale of Tempa test changes pre-and post-treatment and the first week, and the length of the time subscale of Tempa test improvements from pre-intervention to the first week.

**Conclusion:**

Findings underscored the mutual and synergistic benefits of integrating motor and language in stroke rehabilitation. While SLT and AAT were effective when applied independently, their combined application yielded superior outcomes, emphasizing the holistic advantages of integrating these interventions, as supported by existing literature on dual-task rehabilitation strategies.

**Clinical trial registration:**

https://irct.behdasht.gov.ir/search/result?query=IRCT20200114046134N1, IRCT20200114046134N1.

## 1 Introduction

Stroke, a prevalent neurological condition affecting 25% of individuals during their lifetime, ranks as the second leading cause of mortality and the third significant cause of disability worldwide (Feigin et al., 2018; Hajipour et al., 2023; Yaşa et al., 2023). The multifaceted nature of stroke manifests in various disabilities, including motor and language impairments that frequently co-occur. These deficits necessitate a comprehensive rehabilitation approach to address them following brain damage (Anderlini et al., 2019; Ginex et al., 2017; Harnish et al., 2014; Zainaee et al., 2021; Zainaee et al., 2024). Since the early 18th century, neurorehabilitation studies have discovered a tight and mutual relationship between motor and language functions (Harnish et al., 2014), which this interconnectedness highlights the importance of integrated rehabilitation strategies that address both domains to optimize recovery outcomes for stroke patients (Anderlini et al., 2019; Johari et al., 2019a; Nikrah et al., 2023). These neural pathways are primarily positioned within Broca’s area (BA44) of the human cortex, which is crucial for fluency in both movement and language (Anderlini et al., 2019; Arya and Pandian, 2014; Behroozmand et al., 2018). Damage to this region leads to a lack of fluency in the output of both systems, behaviorally observed as simultaneous aphasia and hemiplegia (Anderlini et al., 2019). According to multiple reports, approximately 20 to 38% of post-stroke patients exhibit aphasia (Cichon et al., 2021), with 80% of them experiencing grip and hand extensor muscle deficits (Brihmat et al., 2023). Furthermore, Broca’s aphasia and hemiplegia frequently co-occur at a rate of 80% (Anderlini et al., 2019; Arya and Pandian, 2014; Behroozmand et al., 2018). These findings underscore the positive impact of speech and language Therapy (SLT) and motor training as appropriate interventions for stroke patients’ recovery (Ginex et al., 2017). While neurobehavioral studies have stated the effectiveness of SLT and motor approaches independently, some theories suggest that the neural mechanism responsible for planning hand and speech actions may overlap. Consequently, combining these approaches could yield synergistic effects, potentially enhancing the recovery of stroke patients (Anderlini et al., 2019; Berthier et al., 2023; Cichon et al., 2021; Coppens, 2016; Georgiou and Kambanaros, 2022; Harnish et al., 2014; Heikkinen et al., 2019; Xu et al., 2021; Yaşa et al., 2023). This observation can be attributed to several factors that play a significant role in this process. One such factor is the Mirror Neuron System (MNS), indicating that observing and/or performing arm or mouth/lip movements can stimulate the coactivation of motor and language functions (Anderlini et al., 2019; Arya and Pandian, 2014; Chen et al., 2019; Chen et al., 2015; Pulvermüller and Berthier, 2008). Secondly, brain network functionality can increase the reorganization of motor and language networks (Anderlini et al., 2019; Behroozmand et al., 2018; Chen et al., 2015; Ginex et al., 2017; Hartwigsen and Saur, 2019; Johnson et al., 2022). A recent cohort study discovered a remarkable role of distributed brain network disruption attributed to various impairments such as memory, language, visual, attention, and motor skills following stroke. Findings suggested that dysfunction in network-specific patterns, attributed to a specific behavioral deficit, and loss of interhemispheric communication across a group of areas was related to impairment across numerous behavioral domains (Siegel et al., 2016). Finally, adaptive interactions between language and motor functions occur without competition for resources in neuroplasticity maybe can allowing for synergistic recovery (Coppens, 2016; Harnish et al., 2014; Hartwigsen and Saur, 2019; Primaßin et al., 2015; Pulvermüller and Berthier, 2008). To support this perspective, Gentilucci and Dalla Volta (2008) discovered that language and arm movements are controlled by the same neural system, where grasping influences labial articulation. They proposed that spoken language evolved from facial and manual gestures processed in the Broca area (BA44), responsible for hand and mouth movements (Gentilucci and Dalla Volta, 2008). Harnish et al. (2014) conducted a study aligned with this concept, providing upper-limb exercises without SLT to five chronic stroke patients with upper-extremity hemiparesis and aphasia. They observed synergistic interactions between the motor and language systems during the 6 weeks of intensive motor therapy, highlighting the integration of manual gestures and hand movements with speech and language to facilitate rehabilitation outcomes (Harnish et al., 2014).

Moreover, several studies reported that an intensive rehabilitation approach can yield significant outcomes for stroke patients. In this regard, it has been emphasized that integrating motor and speech training may lead to greater improvements due to the intensity of the tasks involved (Buchwald et al., 2018; Georgiou and Kambanaros, 2022; Hara et al., 2015). Rahimibarghani et al. (2023) recommended that while a weekly schedule of nine-hours of speech therapy would be needed for optimal recovery in stroke patients, combining this with motor tasks, such as motor cortex stimulation, significantly enhances gains in both skills (Rahimibarghani et al., 2023).

Additionally, addressing cognitive impairments, which affect about 70% of stroke patients, is crucial for minimizing long-term disability (Faria et al., 2018). According to a study on functional improvement, task-specific training (TST) combined with cognitive sensorimotor exercise (CSE) significantly improves proprioception, spasticity, and gait speed in stroke patients (Kim and Jang, 2021). Furthermore, employing an integrative approach, such as dual-task training that requires patients to manage two activities simultaneously, increases practice intensity and leads to better performance compared to single-task training (Faria et al., 2018; Kannan et al., 2019; Nikrah et al., 2024; Spanò et al., 2022).

The widespread prevalence of stroke and the increasing number of individuals living with disabilities have underscored the urgent need for effective therapies to accelerate the recovery process. Despite the growing body of research on the interplay between motor and language functions, and their combined effects (Anderlini et al., 2019; Chen et al., 2019; Ginex et al., 2017; Heikkinen et al., 2019; Xu et al., 2021) through utilizing interventions like drugs (Berthier et al., 2023; Cichon et al., 2021; Hartwigsen and Saur, 2019) or techniques such as TDCS (Buchwald et al., 2018; Georgiou and Kambanaros, 2022; Hara et al., 2015; Hartwigsen and Saur, 2019; Yaşa et al., 2023), there is a limitation of studies that have comprehensively investigated the links between these skills coupled with comparing each intervention with combining them behaviorally. Moreover, we did not find any studies that concurrently evaluated both motor and language skills during interventions, along with pre and post-treatment assessments (Xu et al., 2021).

This study aimed to investigate the relationship between language and motor skills and the potential benefits of integrating SLT and motor training in stroke rehabilitation.

This study offers a new perspective and statistical evidence for the “combined assessments and therapist” approach and “the pattern of changing skills during interventions” in the neurorehabilitation of stroke patients. We addressed two research questions:

1. Is there a significant interaction between motor and language skills within-group (those who received only SLT and motor training)?
2. Is SLT and motor training more effective than either intervention when comparing outcomes between groups?

## 2 Methods

### 2.1 Study participants

**Figure 1:**
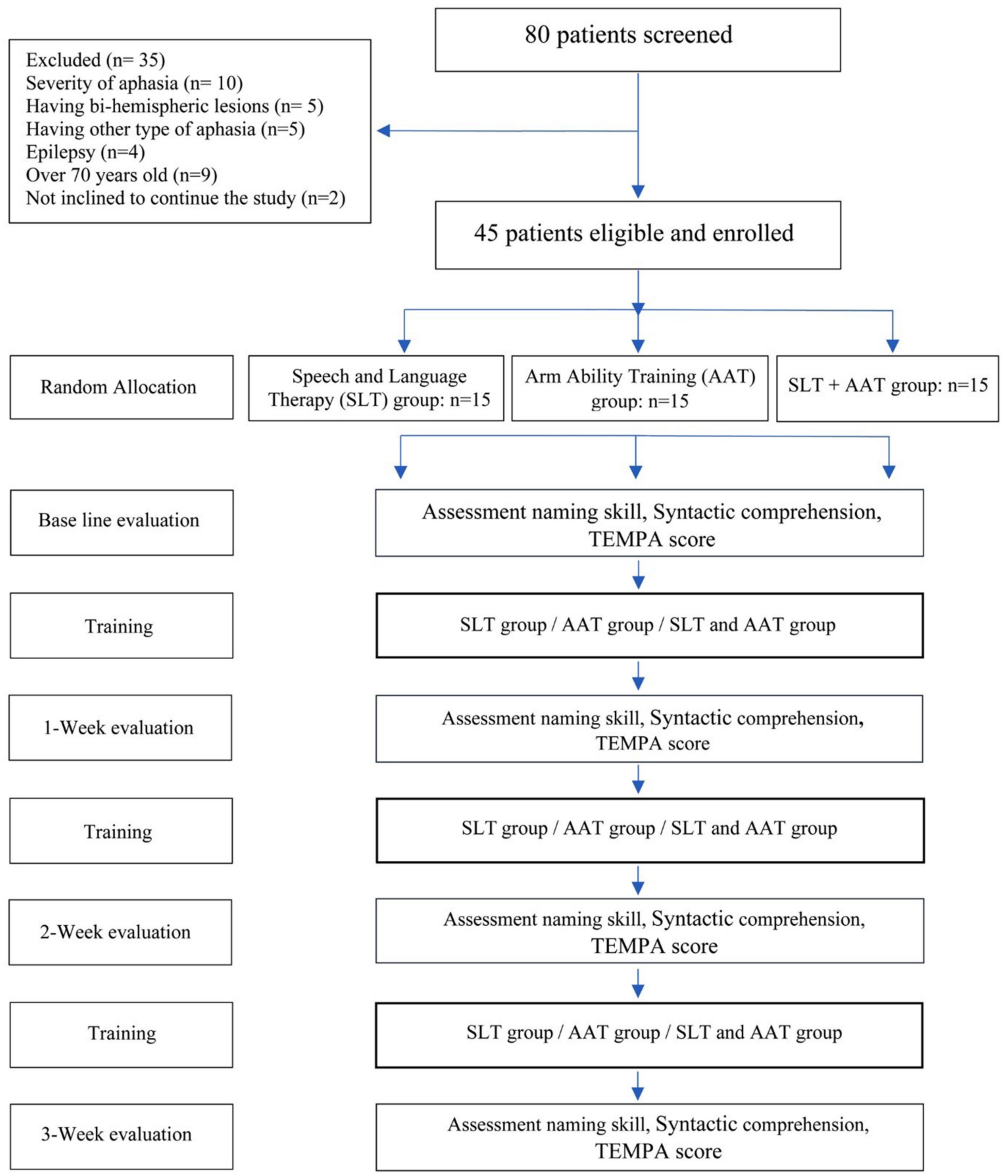
Experimental implementation process of the study.

Data was gathered from 80 individuals at the Stroke Center of Ghaem Hospital in Mashhad, Iran. Following screening, 45 stroke patients (56.25% = 28 males and 17 females) met the eligibility criteria and were enrolled. The exclusion of 35 screened subjects is depicted in Figure 1. The inclusion and exclusion criteria were as follow:

Inclusion criteria:

1. Participants must have experienced their first ischemic, hemorrhagic, or mixed stroke.
2. Present of single or multiple lesions confined to the left frontal lobe, a crucial region for fluency, both sequential action and speech production, and perception, confirmed by Magnetic Resonance Imaging (MRI).
3. Age ranging from 35 to 70 years.
4. Native Persian speakers.
5. Right-handedness before the stroke, examined using the Edinburg Handedness Inventory with a score of 72–100.
6. Mild to moderate severity (Aphasia Quotient = 76–93 to 51–75) of Broca aphasia, determined by a speech therapist using the Persian Western Aphasia Battery (P-WAB).
7. Participants did not have or had mild to moderate dysarthria in order to have ability to answer tests, determined by a speech therapist.
8. Minimum Mini-Mental State Examination (MMSE) score of ≥23 (scores < 23 indicate cognitive impairments).
9. Affirmation of right-hand hemiparesis by physical therapists.
10. Participants were outpatient and in the chronic phase of stroke.

Exclusion criteria:

1. Presence of hemiplegia in the upper limb.
2. Diagnosis of other neurological conditions or psychiatric disorders, beyond the stroke, that could interfere with the treatment, based on medical history.
3. Additional lesions outside the left frontal lobe.
4. Concurrent participation in behavioral, drug, device, or biologic interventions that may impact the study’s outcomes.
5. History of alcohol abuse.
6. Lack of willingness to continue participating in the research project.

### 2.2 Interventions

An open-label, perspective trial was conducted with three groups randomly allocated in a 1:1:1 ratio. The interventions were as follows: SLT group: Participants received regular SLT protocol, incorporating Semantic Feature Analysis (SFA) and the Helm-Estabrooks Language Program for Syntax Stimulation (HELPSS). This selection of intervention was based on the fact that a significant number of Broca’s aphasia cases, attributed to deficits in the left frontal lobe, manifest symptoms like agrammatism and difficulties in word retrieval (den Ouden et al., 2012; Rezaii et al., 2023), Arm Ability Training (AAT) group: Participants underwent motor training using AAT, and SLT + AAT group: Participants received a consecutive combination of these trainings. Both SLT and AAT groups received their interventions for 40 min per session, three days per week, over a period of three weeks. SLT + AAT group received a consecutive combination of SLT and AAT, consisting of nine sessions. Each session comprised a 20-min SLT component followed immediately by a 20-min AAT component. It is important to highlighted that the same therapists always treated the patients. Moreover, after completing the project, for participants in any group who desired to undergo AAT and/or SLT, treatment sessions were held. A flowchart outlining the participant selection process and the study design can be found in Figure 1.

#### 2.2.1 Semantic feature analysis (SFA)

It is an impairment-focused intervention for improving naming abilities in individuals with difficulty, particularly Broca aphasia, agrammatic aphasia, and anomia (Peach and Reuter, 2010). Based on the theory of spreading activation in semantic network processing, SFA works by stimulating the target word with associated semantic features to assist lexical retrieval. The clinician facilitates the patient complete a semantic map with information about the target word, including category, location, action, properties, and associations. If the patient cannot name the picture after completing the map, the clinician provides the name, and the patient repeats it and reviews its associated features (Kristensson et al., 2022).

#### 2.2.2 Helm-Estabrooks language program for syntax stimulation (HELPSS)

This method was developed to promote the syntactic skills of individuals with nonfluent aphasia. The protocol was designed to train 11 different sentences, beginning with the imperative intransitive structures like “sit down” and “watch out,” and progressing to future verb tense, longer active sentences, and wh-questions, and finally, storytelling. A hierarchy of sentence types can be established based on their frequency in language samples collected from aphasic individuals (Goodglass and Berko, 1960; Rezaii et al., 2023).

#### 2.2.3 Arm ability training (AAT)

**Figure 2:**
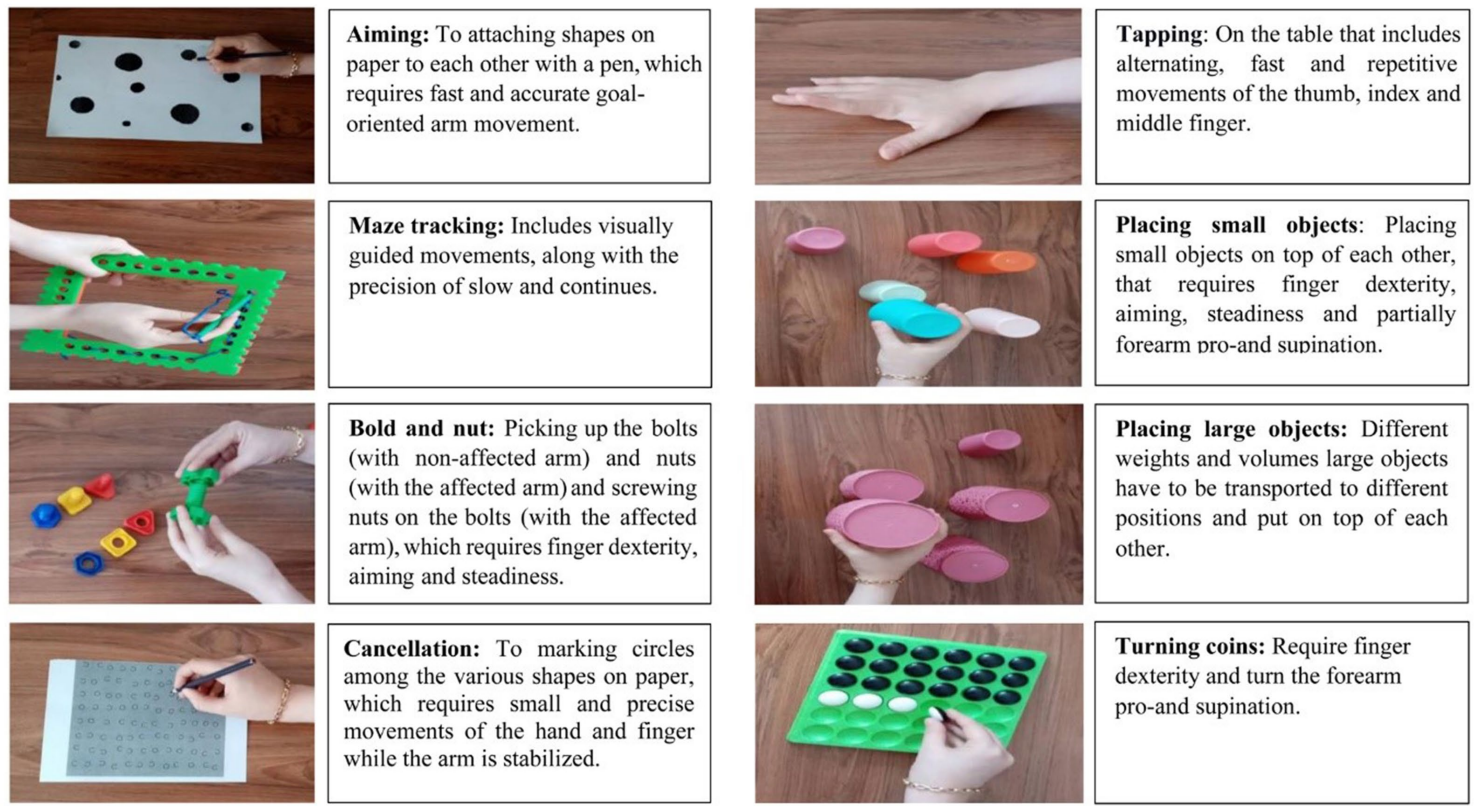
The arm ability training tasks.

AAT is a specialized intervention for arm paresis to enhance speed, strength, accuracy, endurance, and functional activities. The program consists of eight training exercises, as illustrated in Figure 2, focusing on increasing the sensorimotor capabilities of the arm, hand, and fingers, resulting in better motor performance and functional abilities in individuals with arm paresis (Platz et al., 2015; Platz and Lotze, 2018).

### 2.3 Assessments

The evaluation sessions conducted pre-interventions, during the first and second weeks of interventions, and post-intervention in the third week. Each session lasted approximately 60 min for every participant, with breaks provided as necessary. It is important to note that the therapists, administering the interventions and the evaluators, conducting the tests were different individuals. Furthermore, those applying the examinations were unaware of the group assignments of the participants. To mitigate potential learning effects, the evaluation items were not incorporated in the intervention sessions, and participants did not receive any feedback from therapists during the examination process. The picture naming test, syntactic comprehension test, and Test d’Evaluation des Membres Supérieurs de Personnes Âgées (TEMPA) test were used as validated instruments for language and motor assessments, respectively. These tests are commonly utilized in similar research studies (for example, see Desrosiers et al., 1995; Johari et al., 2019b; Saber-Moghadam et al., 2022).

#### 2.3.1 Picture naming test

It consists of 50 objects categorized into animals (12 cases), natural categories (11) and handmade items (27 cases) (Saber-Moghadam et al., 2022). Its validity and reliability were established through a study involving 32 Persian patients aged 40 to 68. The test demonstrates an internal homogeneity coefficient of 0.96 and a test–retest coefficient of 0.87 (*p* < 0.01) (Pour et al., 2014). During the test, participants were presented with each image individually and asked to name it verbally. Their responses were recorded against the target word (Pour et al., 2014; Saber-Moghadam et al., 2022).

#### 2.3.2 Syntactic comprehension

The Farsi version of the Bilingual Aphasia Test (BAT) has been validated and tested for reliability in Persian (Paradis, 1987). This examination includes 30 sentences, with 10 items for each sentence type (negative, subject-topicalized, and object-topicalized). Participants were presented with four pictures and required to select the one that best matched the sentence they heard. The final score was determined by adding the number of correct responses (Johari et al., 2019b).

#### 2.3.3 Motor outcome measures

Motor abilities were examined using the functional rating and length of time subscales from the TEMPA score. This test was employed to determine the strengths and weaknesses of the upper extremity. The validity and the test–retest reliability were established in a study involving 29 patients aged 62 to 82 years (mean age: 70) with various upper limb impairments. The internal correlation coefficients ranged from 0.70 to 0.10 (Desrosiers et al., 1995; Desrosiers et al., 1993). The TEMPA consists of 13 tasks (Desrosiers et al., 1995):

- Pick up and move a jar by the right-hand
- Pick up and move a jar by the left-hand
- Open a jar and take a spoonful of coffee
- Pick up a pitcher and pour water into a glass by the right-hand
- Pick up a pitcher and pour water into a glass by the left-hand
- Unlock a lock and open a pill container
- Write on an envelope and stick on a stamp
- Tie a scarf around one’s neck
- Shuffle and deal playing cards
- Handle coins with the right hand
- Handle coins with the left hand
- Pick up and move small objects by the right hand
- Picking up and moving small objects with the left hand

The functional rating subscale of Tempa test assesses the level of independence in performing these tasks using a 4-point scale (Desrosiers et al., 1995; Desrosiers et al., 1993):

0: Task completed without pause or difficulty.

−1: Some difficulty in completing the entire task.

−2: Great difficulty in completing the entire task.

−3: Patient could not complete the task even with assistance.

The length of time subscale of Tempa test measures the time taken to complete each task in seconds, starting from when the person’s hands leave the table until the task is finished (Desrosiers et al., 1995; Desrosiers et al., 1993).

Trial information was expressed to participants through a language-friendly visual presentation aligned with national guidelines, leading to a comprehensive understanding by both participants and caregivers. This study was approved by the Ethics Committee of Mashhad University of Medical Science (code: IR.MUMS.REC.981582), and all participants provided written informed consent before taking part in the study.

All participants completed the study protocol with robust compliance, and post-trial feedback indicated high satisfaction and a willingness to continue receiving interventions. Notably, there were no reported therapy-related adversities experienced by either participants or caregivers.

### 2.4 Statistical analysis

The normality of quantitative variables was assessed using the Kolmogorov–Smirnov test. Qualitative variables were presented as frequency and percentage, while quantitative variables were reported as mean (standard deviation) or median (1st and 3rd quartiles) for non-normally distributed data. The equality of proportions of qualitative variables among the three groups was evaluated using the Chi-square and Fisher’s exact tests. Analysis of variance was employed to compare the means of normally distributed quantitative variables across the three groups.

For intragroup and intergroup comparisons, the Kruskal-Wallis and Friedman tests were utilized. Pairwise comparisons were conducted using the Mann–Whitney and Wilcoxon rank-sum tests. Bonferroni’s *post hoc* test was applied for further pairwise comparisons. Statistical analyses were performed using SPSS version 26, with a significance level set at *p* < 0.05.

## 3 Results

In order to explore the relationship between motor and language skills and determine the effectiveness of integrating interventions than each intervention, we compared scores test within-group and between group. The clinical and demographic data of 45 participants are illustrated in Table 1.

**Table 1:** Participants characteristics (*N* = 45).

Variables	SLT (*n* = 15)	AAT (*n* = 15)	SLT + AAT (*n* = 15)	Statistic (*p*-value)
Education, *n* (%)				
<High school	9 (60.0)	9 (60.0)	7 (46.7)	χ^2^ = 0.720 *p*-value = 0.698
≥High school	6 (40.0)	6 (40.0)	8 (53.3)	
Gender, *n* (%)				
Male	9 (60)	9 (60)	10 (66.7)	χ^2^ = 0.189 *p*-value = 0.910
Females	6 (40)	6 (40)	5 (33.3)	
Type of stroke (*n*, %)				
Ischemic	6 (40.0)	7 (46.6)	7 (46.6)	Fisher’s Exact Test = 5.491 *p*-value = 0.505
Hemorrhagic	6 (40.0)	6 (40.0)	4 (26.6)	
Mixed	3 (20.0)	2 (13.3)	4 (26.6)	
Age (y), mean ± SD	57.26 ± 9.61	57.66 ± 9.47	57.40 ± 6.79	*F* = 0.008 *p*-value = 0.992
Severity of aphasia AQ, mean ± SD	65.26 ± 17.28	61.33 ± 21.99	63.33 ± 21.26	*F* = 0.639 *p*-value = 0.533
MMSE, mean ± SD	24.53 ± 5.87	25.06 ± 6.09	24.26 ± 6.02	*F* = 0.141 *p*-value = 0.869

AQ, Aphasia Quotient; MMSE, Mini-Mental Scale Examination, Qualitative variables were analyzed using frequency and percentage, and quantitative variables were reported as mean (it was analyzed using the ANOVA). The proportions of qualitative variables among the groups were evaluated using the Chi-square and Fisher’s exact tests. Analysis of variance was employed to compare the means of quantitative variables across the groups. Degree of freedom = 1.

### 3.1 Within-group comparison of scores

**Figure 3:**
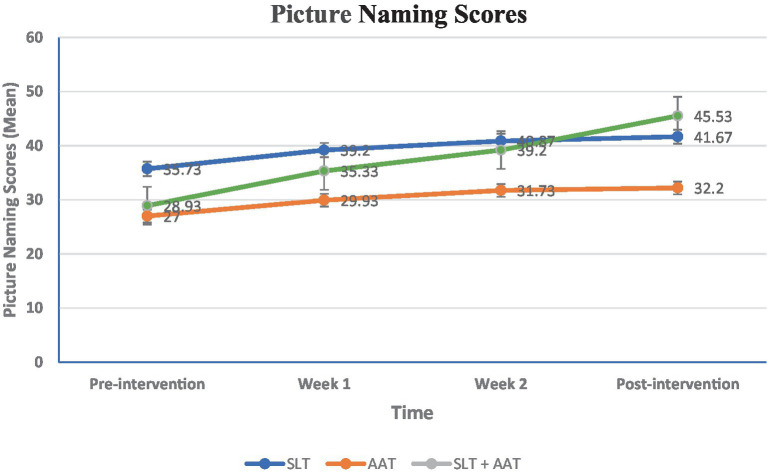
Between and within-groups comparison of scores in different assessment point in picture naming, The variable is expressed as a mean and analyzed using the ANOVA test. Intragroup and intergroup were compared using the Kruskal-Wallis and Friedman tests. Pairwise comparisons were conducted using the Mann–Whitney and Wilcoxon rank-sum tests. Bonferroni’s *post hoc* test was utilized for further pairwise comparisons. A significant difference demonstrated in the first week (*p* = 0.046), and in the pairwise comparison between AAT and SLT + AAT groups (*p* = 0.047) and SLT and SLT + AAT groups (*p* = 0.022).

**Figure 4:**
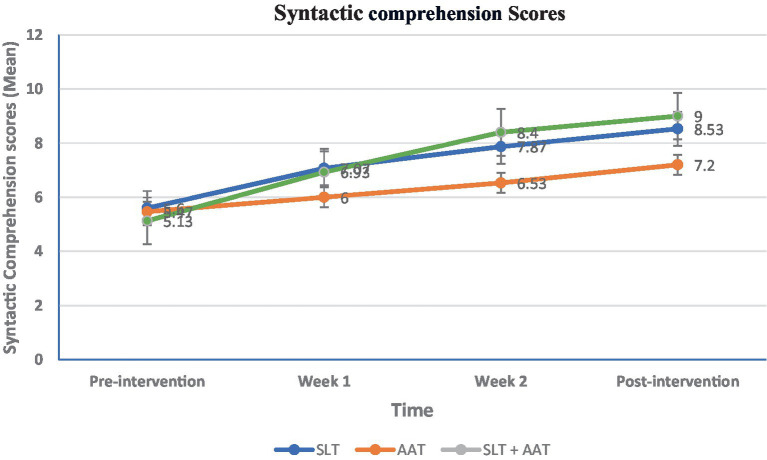
Between and within-groups comparison of scores at different assessment point in syntactic comprehension, The variable is expressed as a mean and analyzed using the ANOVA test. Intragroup and intergroup were compared using the Kruskal-Wallis and Friedman tests. Pairwise comparisons were conducted using the Mann–Whitney and Wilcoxon rank-sum tests. Bonferroni’s *post hoc* test was utilized for further pairwise comparisons. Significant differences were observed in the second and third weeks (*p* = 0.023 and *p* = 0.019, respectively), and the pairwise comparisons were found in the second week between AAT and SLT + AAT groups (*p* = 0.008), and in the third week between AAT and SLT + AAT groups (*p* = 0.007) and SLT and AAT groups (*p* = 0.037).

**Figure 5:**
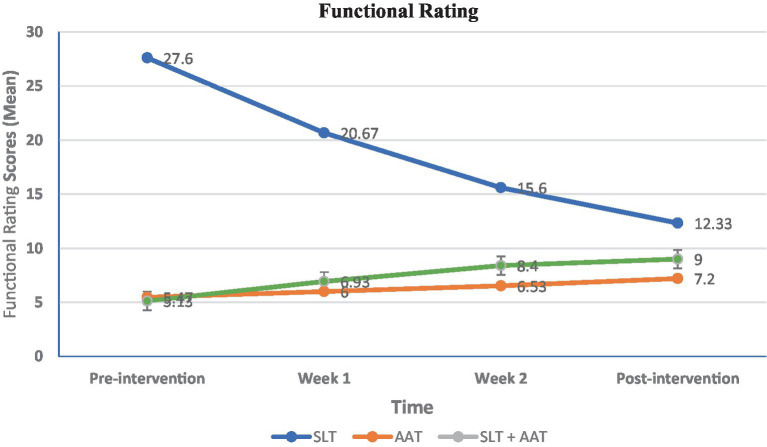
Between and within-groups comparison of scores in different assessment point in functional rating subscale of Tempa assessment as motor evaluation, The variable is expressed as a mean and analyzed using the ANOVA test. Intragroup and intergroup were compared using the Kruskal-Wallis and Friedman tests. Pairwise comparisons were conducted using the Mann–Whitney and Wilcoxon rank-sum tests. Bonferroni’s *post hoc* test was utilized for further pairwise comparisons. Significant differences were exhibited pre-intervention (*p* = 0.010), the first week (*p* = 0.023), and the third week (*p* = 0.001), and pairwise comparisons revealed significant differences between SLT and SLT + AAT groups at pre-intervention (*p* = 0.003), in the first week (*p* = 0.006), and SLT and SLT + AAT groups (*p* = 0.001) and SLT and AAT groups (*p* = 0.002) in the third week.

**Figure 6:**
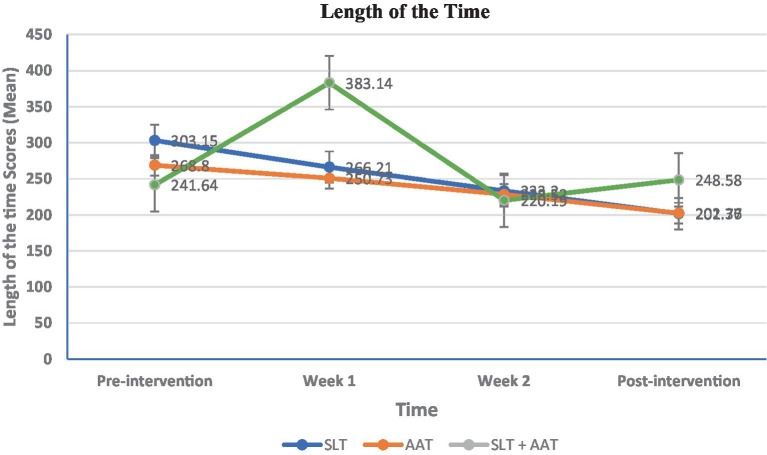
Between and within-groups comparison of scores in different assessment point in length of time subscale of Tempa assessment as motor evaluation, The variable is expressed as a mean and analyzed using the ANOVA test. Intragroup and intergroup were compared using the Kruskal-Wallis and Friedman tests. Pairwise comparisons were conducted using the Mann–Whitney and Wilcoxon rank-sum tests. Bonferroni’s *post hoc* test was utilized for further pairwise comparisons. Significant differences were noted at pre-intervention (*p* = 0.001) and in the first week (*p* = 0.011). The pairwise comparisons exhibited significant differences at pre-intervention between SLT and SLT + AAT groups (*p* < 0.001), in the first week between SLT and SLT + AAT groups (*p* = 0.004), and AAT and SLT + AAT groups (*p* = 0.036).

In terms of language examinations, significant differences were observed in pre-and post-intervention picture naming scores within SLT group (*p* = 0.024), AAT group (*p* = 0.003), and SLT + AAT group (*p* < 0.001) (refer to Table 2 and Figure 3). The growth rates (percentage differences between post-and pre-intervention scores divided by pre-intervention scores) for SLT, AAT, and SLT+ AAT groups were 16.62, 48.14, and 57.37, respectively. Refer to Table 2 for detailed insights into the score comparisons within groups for this outcome. Additionally, all groups displayed significant disparities (*p* < 0.001) in pre-and post-intervention syntactic comprehension scores (see Table 3 and Figure 4). The growth rates for SLT, AAT, and SLT+ AAT groups were 52.32, 31.62, and 75.43, respectively. More detailed data on the score comparisons within groups for this measure can be found in Table 3. Regarding motor training, significant differences in pre-and post-intervention functional rating subscale of Tempa test scores were noted across all groups (*p* < 0.001) (refer to Table 4 and Figure 5). The growth rates for SLT, AAT, and SLT+ AAT groups were −55.32, −62.93, and −60.87, respectively. Further insight into the comparisons within groups for this subcategory is available in Table 4. Furthermore, notable differences in the duration between pre-and post-interventions were observed in SLT group (*p* < 0.001), AAT group (*p* < 0.001), and SLT + AAT group (*p* = 0.021) (as illustrated in Table 5 and Figure 6). The growth rates for Groups A, B, and C were − 32.53, −15.24, and 2.87, respectively. Additional information on the comparisons within groups for this metric can be found in Table 5.

**Table 2:** The picture naming profile of participants at different timepoints.

Groups	Time Mean ± SD Median (Q1, Q3)				Pairwise comparison (within group)
	Pre-intervention	Week 1	Week 2	Post-intervention	
Speech and language therapy (SLT)	35.73 ± 20.35 50 (15, 50)	39.2 ± 17.08 50 (28, 50)	40.87 ± 15.62 50 (35, 50)	41.67 ± 14.72 50 (35, 50)	0.001 Pre-intervention vs. Week 1: 0.322 Pre-intervention vs. Week 2: 0.066 Pre vs. post-intervention: 0.024 Week 1 vs. Week 2: 0.396 Week 1 vs. post-intervention: 0.203 Week 2 vs. post-intervention: 0.671
Arm ability training (AAT)	27 ± 18.51 38 (5, 40)	29.93 ± 19.14 40 (5, 46)	31.73 ± 21.11 40 (5, 50)	32.2 ± 20.72 40 (10, 50)	0.0002 Pre-intervention vs. Week 1: 0.138 Pre-intervention vs. Week 2: 0.004 Pre vs. post intervention: 0.003 Week 1 vs. Week 2: 0.157 Week 1 vs. post-intervention: 0.138 Week 2 vs. post-intervention: 0.944
SLT + AAT	28.93 ± 15.97 35 (20, 40)	35.33 ± 14.94 42 (33, 45)	39.2 ± 13.39 45 (24, 50)	45.53 ± 7.06 50 (45, 50)	(*p* < 0.0001) Pre-intervention vs. Week 1: 0.034 Pre-intervention vs. Week 2: (*p* < 0.0001) Pre vs. post intervention: (*p* < 0.0001) Week 1 vs. Week 2: 0.034 Week 1 vs. post-intervention: (*p* < 0.0001) Week 2 vs. post-intervention: 0.120
Pairwise comparison (between groups)	0.059	0.046 A vs. B: 0.763 A vs. C: 0.022 B vs. C: 0.047	0.369	0.239	

Significant at 0.05, Data was analyzed using ANOVA (mean ± SD) and Kruskal-Wallis (Medians) tests, Intragroup and intergroup were compared using the Kruskal-Wallis and Friedman tests. Pairwise comparisons were conducted using the Mann–Whitney and Wilcoxon rank-sum tests. Bonferroni’s post hoc test was utilized for further pairwise comparisons. Degree of freedom = 1.

**Table 3:** The syntactic comprehension profile of participants at different timepoints.

Groups	Time Mean ± SD Median (Q1, Q3)				Pairwise comparison (within group)
	Pre-intervention	Week 1	Week 2	Post-intervention	
Speech and language therapy (SLT)	5.6 ± 1.45 5 (5, 7)	7.07 ± 2.19 6 (5, 10)	7.87 ± 2.13 8 (6, 10)	8.53 ± 2.07 10 (7, 10)	(*p* < 0.0001) Pre-intervention vs. Week 1: 0.013 Pre-intervention vs. Week 2: (*p* < 0.0001) Pre vs. post-intervention: (*p* < 0.0001) Week 1 vs. Week 2: 0.179 Week 1 vs. post-intervention: 0.024 Week 2 vs. post-intervention: 0.358
Arm ability training (AAT)	5.47 ± 1.25 5 (4, 6)	6 ± 1.31 6 (5, 7)	6.53 ± 1.64 7 (5, 7)	7.2 ± 1.61 7 (6, 8)	(*p* < 0.0001) Pre-intervention vs. Week 1: 0.157 Pre-intervention vs. Week 2: 0.002 Pre vs. post-intervention: (*p* < 0.0001) Week 1 vs. Week 2: 0.104 Week 1 vs. post-intervention: 0.001 Week 2 vs. post-intervention: 0.066
SLT + AAT	5.13 ± 0.74 5 (5, 5)	6.93 ± 1.22 7 (6, 8)	8.4 ± 1.72 8 (7, 10)	9 ± 1.69 10 (8, 10)	(*p* < 0.0001) Pre-intervention vs. Week 1: 0.040 Pre-intervention vs. Week 2: (*p* < 0.0001) Pre vs. post-intervention: (*p* < 0.0001) Week 1 vs. Week 2: 0.009 Week 1 vs. post-intervention: 0.001 Week 2 vs. post-intervention: 0.437
Pairwise comparison (between groups)	0.605	0.155	0.023 A vs. B: 0.052 A vs. C: 0.480 B vs. C: 0.008	0.019 A vs. B: 0.037 A vs. C: 0.546 B vs. C: 0.007	

Significant at 0.05, Data was analyzed using ANOVA (mean ± SD) and Kruskal-Wallis (Medians) tests. Intragroup and intergroup were compared using the Kruskal-Wallis and Friedman tests. Pairwise comparisons were conducted using the Mann–Whitney and Wilcoxon rank-sum tests. Bonferroni’s post hoc test was utilized for further pairwise comparisons. Degree of freedom = 1.

**Table 4:** The functional rating subscale of Tempa test at different timepoints.

Groups	Time Mean ± SD Median (Q1, Q3)				Pairwise comparison (within group)
	Pre-intervention	Week 1	Week 2	Post-intervention	
Speech and language therapy (SLT)	27.60 ± 4.48 28 (25, 31)	20.67 ± 4.64 21 (17, 24)	15.60 ± 3.27 15 (13, 17)	12.33 ± 2.26 13 (12, 13)	(*p* < 0.0001) Pre-intervention vs. Week 1: 0.028 Pre-intervention vs. Week 2: (*p* < 0.0001) Pre vs. post intervention: 0.000 Week 1 vs. Week 2: 0.034 Week 1 vs. post-intervention: (*p* < 0.0001) Week 2 vs. post-intervention: 0.05
Arm ability training (AAT)	5.47 ± 1.25 5 (4, 6)	6 ± 1.31 6 (5, 7)	6.53 ± 1.64 7 (5, 7)	7.2 ± 1.61 7 (6, 8)	(*p* < 0.0001) Pre-intervention vs. Week 1: 0.034 Pre-intervention vs. Week 2: (*p* < 0.0001) Pre vs. post-intervention: (*p* < 0.0001) Week 1 vs. Week 2: 0.034 Week 1 vs. post-intervention: (*p* < 0.0001) Week 2 vs. post-intervention: 0.034
SLT + AAT	5.13 ± 0.74 5 (5, 5)	6.93 ± 1.22 7 (6, 8)	8.4 ± 1.72 8 (7, 10)	9 ± 1.69 10 (8, 10)	(*p* < 0.0001) Pre-intervention vs. Week 1: 0.013 Pre-intervention vs. Week 2: (*p* < 0.0001) Pre vs. post-intervention: (*p* < 0.0001) Week 1 vs. Week 2: 0.120 Week 1 vs. post-intervention: (*p* < 0.0001) Week 2 vs. post-intervention: 0.028
Pairwise comparison (between groups)	0.010 A vs. B: 0.058 A vs. C: 0.003 B vs. C: 0.263	0.023 A vs. B: 0.193 A vs. C: 0.006 B vs. C: 0.148	0.080	0.001 A vs. B: 0.002 A vs. C: 0.001 B vs. C: 0.784	

Significant at 0.05, Data was analyzed using ANOVA (mean ± SD) and Kruskal-Wallis (Medians) tests. Intragroup and intergroup were compared using the Kruskal-Wallis and Friedman tests. Pairwise comparisons were conducted using the Mann–Whitney and Wilcoxon rank-sum tests. Bonferroni’s post hoc test was utilized for further pairwise comparisons. Degree of freedom = 1.

**Table 5:** The length of the time subscale of Tempa test at different timepoints.

Groups	Time Mean ± SD Median (Q1, Q3)				Pairwise comparison (within group)
	Pre-intervention	Week 1	Week 2	Post-intervention	
Speech and language therapy (SLT)	303.15 ± 38.98 311.13 (276.33, 337.08)	266.21 ± 34.02 277.28 (235.35, 294.91)	233.2 ± 23.2 239.48 (211.94, 250.65)	201.77 ± 17.5 204.52 (187.89, 213.05)	(*p* < 0.0001) Pre-intervention vs. Week 1: 0.034 Pre-intervention vs. Week 2: (*p* < 0.0001) Pre vs. post-intervention: (*p* < 0.0001) Week 1 vs. Week 2: 0.034 Week 1 vs. post-intervention: (*p* < 0.0001) Week 2 vs. post-intervention: 0.034
Arm ability training (AAT)	268.8 ± 20.75 271.11 (257.74, 281.96)	250.73 ± 16.18 246.21 (241.42, 264.83)	228.52 ± 14.03 227.83 (217.24, 239.63)	202.36 ± 16.41 200.01 (198.73, 210.48)	(*p* < 0.0001) Pre-intervention vs. Week 1: 0.120 Pre-intervention vs. Week 2: (*p* < 0.0001) Pre vs. post-intervention: 0.00 (*p* < 0.0001) 0 Week 1 vs. Week 2: 0.016 Week 1 vs. post-intervention: (*p* < 0.0001) Week 2 vs. post-intervention: 0.034
SLT + AAT	241.64 ± 45.64 231.4 (206.59, 278.24)	383.14 ± 612.51 224.43 (192.81, 241.09)	220.19 ± 26.01 220.16 (206.22, 230.42)	248.58 ± 176.72 204.15 (187.91, 224.49)	0.021 Pre-intervention vs. Week 1: 0.157 Pre-intervention vs. Week 2: 0.157 Pre vs. post-intervention: 0.002 Week 1 vs. Week 2: (*p* < 0.0001) Week 1 vs. post-intervention: 0.090 Week 2 vs. post-intervention: 0.090
Pairwise comparison (between groups)	0.001 A vs. B: 0.059 A vs. C: 0.000 B vs. C: 0.071	0.011 A vs. B: 0.42 A vs. C: 0.004 B vs. C: 0.036	0.164	0.909	

Significant at 0.05, Data was analyzed using ANOVA (mean ± SD) and Kruskal-Wallis (Medians) tests, Intragroup and intergroup were compared using the Kruskal-Wallis and Friedman tests. Pairwise comparisons were conducted using the Mann–Whitney and Wilcoxon rank-sum tests. Bonferroni’s post hoc test was utilized for further pairwise comparisons. Degree of freedom = 1.

### 3.2 Between-group comparison of scores

Concerning language skills, the picture naming test exhibited a significant difference only in the first week (*p* = 0.046), and in the pairwise comparison between AAT and SLT + AAT groups (*p* = 0.047) and SLT and SLT + AAT groups (*p* = 0.022). As demonstrated in Table 2 and Figure 3, SLT group showed better performance in this skill compared to other groups. Moreover, significant differences in syntactic comprehension scores were observed in the second and third weeks (*p* = 0.023 and *p* = 0.019, respectively). Noteworthy differences in the pairwise group comparisons were found in the second week between AAT and SLT + AAT groups (*p* = 0.008), and in the third week between AAT and SLT + AAT groups (*p* = 0.007) and SLT and AAT groups (*p* = 0.037). From the data in Table 3 and Figure 4, it is apparent that in the second week SLT + AAT group had better function compered to AAT group and in the third week the performance of SLT and SLT + AAT groups was roughly similar and better than that of AAT group (refer to Table 3 and Figure 4).

In the realm of motor skills, a downward trend in Functional Rating subscale of Tempa test scores displayed significant differences between pre-intervention (*p* = 0.010), the first week (*p* = 0.023), and the third week (*p* = 0.001). Pairwise group comparisons revealed significant differences between SLT and SLT + AAT groups at pre-intervention (*p* = 0.003) and in the first week (*p* = 0.006), which SLT + AAT group in both point time exhibited better function than SLT group. Likewise, SLT and SLT + AAT groups (*p* = 0.001) and SLT and AAT groups (*p* = 0.002) demonstrated significant differences in the third week, which AAT and SLT + AAT groups perform better than AAT group (refer to Table 4 and Figure 5). Additionally, in the examination of length of time subscale of Tempa test, significant differences indicative of a downward trend was noted at pre-intervention (*p* = 0.001) and in the first week (*p* = 0.011). Pairwise group comparisons exhibited significant differences at pre-intervention between SLT and SLT + AAT groups (*p* < 0.001) that SLT + AAT group exhibited better function that SLT group. Additionally, there were significant difference in the first week between SLT and SLT + AAT groups (*p* = 0.004) and AAT and SLT + AAT groups (*p* = 0.036), which SLT + AAT group had better performance than that of SLT and AAT groups (as demonstrated in Table 5 and Figure 6).

## 4 Discussion

In this study, we investigated the interplay between motor and language skills by comparing outcomes within-group and evaluating the impacts of consecutive combination of SLT and AAT compared to each intervention separately in individuals with Broca’s aphasia. Consistent with previous studies (Hybbinette et al., 2021; Xu et al., 2021), findings support the hypothesis that positive correlations exist between motor and language functions in post-stroke patients. Additionally, the results suggest that integrating these interventions may yield greater benefits than either intervention.

### 4.1 The interactions of motor and language skills

Preliminary findings from comparing within-group outcomes (only SLT and AAT), revealed that SLT, which is effective in enhancing language skills, also positively impacts motor skills. Similarly, AAT, typically associated with improving hand motor functions, was found to promote language abilities. These results may be interpreted in light of Vainio et al.’s (2015) findings, which suggest that the group of neurons in the premotor area F5, involved in manual grasp and mouth motor actions, are homologous to human Broca’s area. This implies that manual actions were likely initially connected to mouth/oral actions, and speech skills emerged from these connections (Vainio et al., 2015). Consequently, speech, oral, and hand gestures are associated anatomically, functionally, and evolutionarily (Hemmati et al., 2023; Sabermoghadam and Sarvghadi, 2023; Vainio et al., 2015). However, it remains unclear whether this link extends beyond mouth and hand motor functions to other motor abilities, such as leg movements (Vainio et al., 2015).

#### 4.1.1 The effect of AAT on picture naming and syntactic comprehension

Picture naming and syntactic comprehension evaluations pointed out that AAT without SLT in AAT group could activate language processes, leading to improvements after three weeks. Recent neuropsychological research has mentioned that language and motor processing are not distinct modules at the cortical level, rather, they are interconnected and function in parallel (Anderlini et al., 2019; Harnish et al., 2014; Hybbinette et al., 2021; Pulvermüller and Berthier, 2008; Xu et al., 2021). Current findings support these statements and offer an explanation for this phenomenon. The trend of scores on the picture naming scale in AAT group demonstrated a significant difference between pre-intervention (27 ± 18.51) and post-intervention (32.2 ± 20.72), indicating that AAT without SLT can improve picture naming ability. In agreement with these results, Chen et al. (2015) emphasized that motor training and hand exercises can facilitate language recovery. In their study, participants were divided into two groups, followed two distinct protocols: protocol A involved observing hand movements combined with word repetition, while Protocol B involved observing static objects combined with word repetition. The findings revealed that protocol A exhibited greater improvement in language abilities through increased activation of mirror neurons (Chen et al., 2015). Consistence with a growing body of evidence (Buchwald et al., 2018; Chen et al., 2019; Chen et al., 2015; Hara et al., 2015; Harnish et al., 2014; Primaßin et al., 2015), patients in AAT group also showed improvement in syntactic comprehension after receiving AAT without SLT, where the trend of scores at pre-intervention (5.47 ± 1.25) and post-intervention (7.2 ± 1.61) was significant. Arya and Pandian (2014) reported similar results in a case report, suggesting that hand exercises may stimulate mirror neurons that overlap with language regions in the brain, thereby promoting language recovery. Their exercises included lifting a glass and bringing it up to the mouth, turning round cans, lifting a cube block, cleaning the table using a wrist-duster, grabbing, dropping a softball, and tying U-shaped clamps (Arya and Pandian, 2014).

Additionally, Xu et al. (2021) administered a behavioral cross-sectional study, that investigated the associations between Upper Extremity (UE) motor function and aphasia in 435 stroke patients. They hypothesized that individuals with aphasia would exhibit poorer UE motor functions compared to those without aphasia and that UE motor status would be positively correlated with language abilities. Their findings confirmed a positive link between four language dimensions; including spontaneous speech, comprehension, repetition, and naming, with UE motor function. Specifically, spontaneous speech was identified as a motor predictor, showing the strongest correlation with UE motor statues, while comprehension showed the weakest association with UE motor skill (Xu et al., 2021). In contrast, although this study also found a positive interplay between motor and language functions, AAT led to greater improvements in syntactic comprehension (*p* < 0.0001) compared to than picture naming skill (*p* = 0.003) after a three-week intervention. This discrepancy could be attributed to several factors, such as differences in study design, participant characteristics, and specific intervention protocols.

#### 4.1.2 The effect of SLT on Tempa

The functional rating and length of time from the Tempa test showed a similar pattern to the effect of AAT on language functions. There was a positive correlation between language and motor domains, suggesting that SLT can improve hand motor. The findings showed significant differences in functional rating (27.60 ± 4.48 to 12.33 ± 2.26) and length of time (303.15 ± 38.98 to 201.77 ± 17.5) subscales of Tempa test for patients in SLT group at the pre-intervention and post-intervention assessments. In contrast to these findings, Primaßin et al. (2015) suggested a positive link and no “fight for resources” between the motor and language domains during recovery. However, in contrast to results of this study, they suggested some hints of additive effects. Their study involved four patients with different motor and language functions: Patient 1 (Base: M+/L+), Patient 2 (Base: M−/L+), Patient 3 (Base: M+/L−) and Patient 4 (Base: M−/L−). Among them, only patient 3, who positively responded to motor therapy, showed significant improvement in language functions, which may have facilitated a favorable response to language therapy. Patient 2, despite showing significant improvements in motor functions, did not exhibit measurable improvements in the language domain. Similarity, patients 1 and 4, who did not benefit from the intensive motor therapy program, also did not show significant improvement in language skills. The authors assumed that positive therapy-induced motor recovery is essential for recovering language skills through language therapy (Primaßin et al., 2015).

### 4.2 Comparing consecutive combination of SLT and AAT with each intervention

Secondary findings indicate that when comparing groups, SLT and SLT + AAT groups or AAT and SLT + AAT groups, combining motor and language interventions may be more effective than using them individually. In parallel with this study, Can Yaşa et al. (2023) compare the effect of (a) SLT through Semantic Feature Analysis (SFA) alone, (b) Transcranial Magnetic Stimulation (TMS) alone, and (c) consecutive SLT + TMS on improving language skills, naming, and quality of life on Broca’s aphasia and control group (10 participants in each group). Their analysis revealed that while TMS and SLT separately were effective, individuals in the group that received the combined SLT + TMS interventions demonstrated greater improvements in speech fluency, repetition, and naming scores from pre-test to post-test (Yaşa et al., 2023). Moreover, Chiaramonte et al. (2024) in their study highlighted the crucial role of intensive therapy in the rehabilitation of stroke patients for improving outcomes. They implemented a comprehensive and synergistic rehabilitative strategy through incorporating three distinct kinds of approaches: (1) goal-oriented training, (2) proprioceptive training, and (3) dual task training. The researchers found that integrating these techniques with traditional treatment (postural and core exercises and gait training) enabled patients to recover independence and balance, while also reducing the risk of falls, which is significant for their caregivers as well (Chiaramonte et al., 2024).

#### 4.2.1 Comparing consecutive combination of SLT and AAT with SLT

In the current study, subjects in SLP and SLP + AAT groups experienced a significant change in the picture naming test during the first week. These results recommended that while language training alone can effectively increase picture naming, combining interventions may result in even greater improvements. This aligns with the findings of Ginex et al. (2017), who examined the effectiveness of SLT and motor training in a retrospective cohort study. They reported that 35% of patients exhibited enhancements in both motor and language functions, indicating that the synergistic effect of combining these approaches can promote overall efficiency (Ginex et al., 2017). However, when comparing the two groups, in terms of the syntactic comprehension tests, no significant differences were observed, which is consistent with previous studies (Gonzalez et al., 2021; Lahiri et al., 2021). This highlighted the complexity of stroke rehabilitation, influenced by demographic variables like age (Meinzer et al., 2012) and various factors such as treatment techniques and lesion characteristics (Hodics et al., 2006). In a related study, Moulton et al. (2019) explored motor and language outcomes in mild-to-severe stroke patients, emphasizing the importance of white matter in the brain for these functions. They noted that although long-term upper limb outcomes are closely linked to the preservation of the corticospinal tract (CST), language outcomes rely on a broader network of white matter fasciculi, including the arcuate fasciculus (AF), inferior fronto-occipital fasciculus (IFOF), inferior longitudinal fasciculus (ILF), and uncinate fasciculus. Furthermore, language outcomes are influenced by factors such as age and initial aphasia severity (Moulton et al., 2019).

#### 4.2.2 Comparing the efficacy of consecutive combination of SLT and AAT with AAT

Participants in AAT and SLP + AAT groups indicated significant differences in the length of time subscale of Tempa test only during the first week. This finding pointed out that the most substantial changes occurred when SLT and AAT were applied together, rather than with AAT alone. This aligns with a study by Chen et al. (2019), which confirmed that combining hand movements and word repetition was more effective in promoting language recovery than separate interventions. In their study, patients were divided into three groups: speech therapy, hand exercise observation with word repetition, and stimulus observation without hand movement. The group that observed hand movements and repeated words exhibited the greatest recovery in language skills, attributed to increased activation of mirror neurons that stimulate language regions in the brain (Chen et al., 2019). A stroke may disrupt the shared pathways of language and motor processing in the brain, which could explain why many stroke patients experience concurrent difficulties in both motor and speech abilities. However, further research is required to explore these connections and their underlying mechanisms more precisely. Additionally, this study did not find any significant differences in the functional rating subscales of Tempa test. It has been suggested that while shared plasticity mechanisms likely exist between hand motor and language skills, individual variability can significantly impact the extent and timing of recovery in these domains (Hybbinette et al., 2021).

## 5 Conclusion

Current findings indicate that motor and language skills have positive interactions and amelioration effects. Therefore, the presence of language deficits should prompt therapists to consider motor training, and vice versa in stroke neurorehabilitation. While each type of intervention can be effective individually, combining them may result in greater improvements in both motor and language skills. Given that many stroke survivors, especially those with Broca’s aphasia, suffer from co-occurring aphasia and motor dysfunctions, their neurorehabilitation program should incorporate both SLT and motor training. By integrating SLT and AAT approaches, therapists can enhance their effectiveness, as evidenced by the improved outcomes observed in this study when consecutive combination of SLT and AAT were administered. Particularly noteworthy is the high prevalence of dysarthric speakers’ post-stroke, ranging from 41.5 to 53%. Treating dysarthria alongside aphasia and motor disabilities through intensive strategies in the rehabilitation process can significantly enhance recovery after stroke (Brady et al., 2011; Chiaramonte and Vecchio, 2021). It is recommended that clinicians adopt a holistic approach to address both language and motor challenges in stroke survivors. By combining interventions and considering each patient’s unique needs, therapists can optimize the recovery process and restore communication abilities.

### 5.1 Study limitations and direction for the future study

Despite the present study suggesting promising evidence, several limitations should be acknowledged. First, we did not access to functional Magnetic Resonance Imaging (fMRI) and positron emission tomography (PET) to examine the neural mechanisms underlying motor and language processes and connectivity analyses, which were required to map the network structure between activated areas. These methods are typically the preferred standard for comprehensive examinations in neurological patients. Second, while efforts were made to recruit as many participants as possible within the constraints of time and resources, a larger sample size, more intervention sessions, and extended follow-up with patients would have been preferable. Nonetheless, we believe that these findings still provide valuable preliminary insights that can guide future research. Therefore, it is important to interpret these findings with caution. Researchers conducting similar studies in the future should take this limitation in to account.

## Data Availability

The original contributions presented in the study are included in the article/supplementary material, further inquiries can be directed to the corresponding author.
